# Spinal Intradural, Extramedullary Ependymoma with Astrocytoma Component: A Case Report and Review of the Literature

**DOI:** 10.1155/2016/3534791

**Published:** 2016-05-29

**Authors:** Gene M. Weinstein, Knarik Arkun, James Kryzanski, Michael Lanfranchi, Gaurav K. Gupta, Harprit Bedi

**Affiliations:** ^1^Department of Radiology, Tufts Medical Center, Boston, MA, USA; ^2^Department of Pathology and Laboratory Medicine, Tufts Medical Center, Boston, MA, USA; ^3^Department of Neurosurgery, Tufts Medical Center, Boston, MA, USA; ^4^Department of Radiology, Massachusetts General Hospital, Boston, MA, USA

## Abstract

Ependymomas are common spinal lesions, with the vast majority arising in an intramedullary location. Several cases have been described in the literature of ependymomas in an intradural, extramedullary location. The authors present a case of a 56-year-old female who presented with several weeks of lower back pain and weakness. MRI revealed an intradural, extramedullary enhancing mass at L1-L2. The mass was successfully resected surgically. Pathologic evaluation revealed a low grade glioma with components of both ependymoma and pilocytic astrocytoma with MUTYH G382D mutation. Extramedullary ependymomas are very rare tumors. To the authors' knowledge, this is the first case of ependymoma/astrocytoma collision tumors described in an extramedullary location.

## 1. Introduction

Most adult ependymomas are spinal lesions, with the vast majority arising in an intradural, intramedullary location [1]. Intradural, extramedullary ependymomas are exceptionally rare tumors that have been reported in the literature [2, 3]. We report a case of an intradural, extramedullary ependymoma with an astrocytoma component. To the best of our knowledge, this has not been described in the literature before. Both the imaging and pathologic features are described here.

## 2. Case Report

A 56-year-old female presented to an outside hospital for several months of low back pain, bilateral hip pain, and leg pain/weakness. She subsequently had an episode of severe low back pain that resulted in difficulty breathing and loss of consciousness. Review of systems revealed several episodes of incontinence. Physical exam was remarkable for 5/5 strength in all extremities, 2+ reflexes in the lower extremities, and a negative Babinski sign.

**Figure 1:**
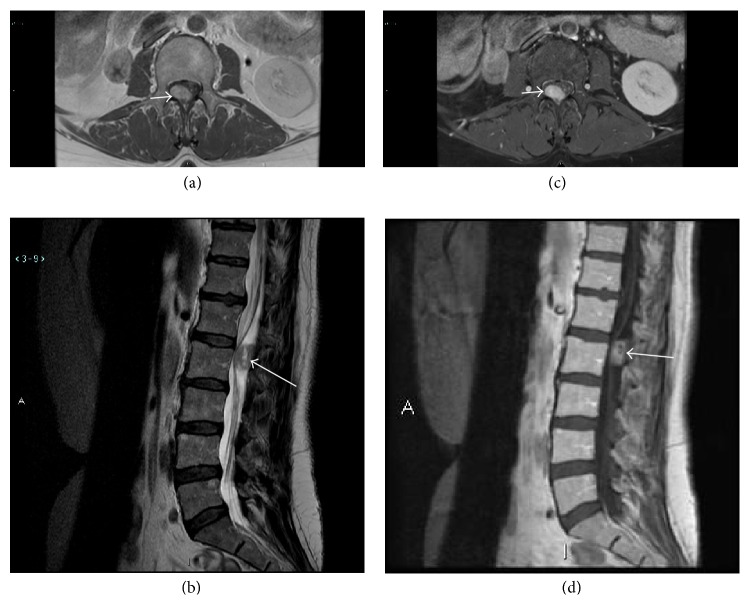
Axial T1 (a) and sagittal T2 (b) sequence shows a round mass (arrows) posterior to the cauda equina at L1-L2 that is hypointense on T1WI and heterogeneously hyperintense on T2WI. Axial T1 FS postcontrast (c) and sagittal T1 postcontrast (d) sequence shows an enhancing intradural, extramedullary mass (arrows) posterior to the cauda equina at L1-L2.

Magnetic resonance imaging of the lumbar spine performed with and without intravenous contrast revealed a 2.1 cm × 1.5 cm × 1.3 cm intradural, extramedullary mass at L1-L2 which compressed the filum terminale. Precontrast MRI showed intrinsic T1 hypointensity and heterogeneous T2 hyperinstensity (Figures 1(a) and 1(b)). Postcontrast imaging showed homogenous postcontrast enhancement (Figures 1(c) and 1(d)). The presumed diagnosis was schwannoma.

**Figure 2:**
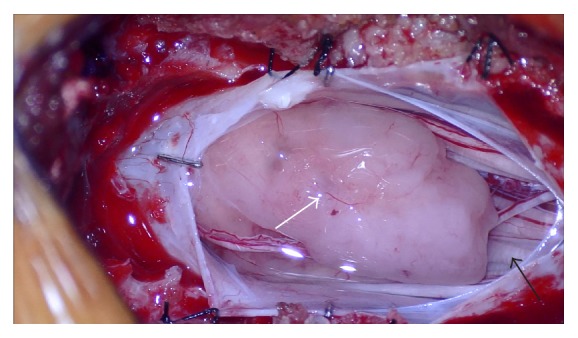
Intraoperative photo shows a tan, fleshy tumor (white arrow) within the thecal sac, clearly outside the cauda equina (black arrow).

Given the patient's symptoms, she underwent elective operative resection of the mass. A posterior L1-L2 laminectomy was performed. A tan, fleshy intradural tumor was identified (Figure 2). Most of the cauda equina was ventral to the tumor. Several small vessels were seen feeding the tumor, which were coagulated. Several small nerve rootlets were also seen leading into the tumor and the tumor had grown around one of the rootlets. The tumor did not appear to arise from any one particular rootlet and was distinct from the filum terminale. The nerve rootlets were dissected free of the tumor and the tumor was removed en bloc. The patient tolerated the procedure well and there were no complications.

**Figure 3:**
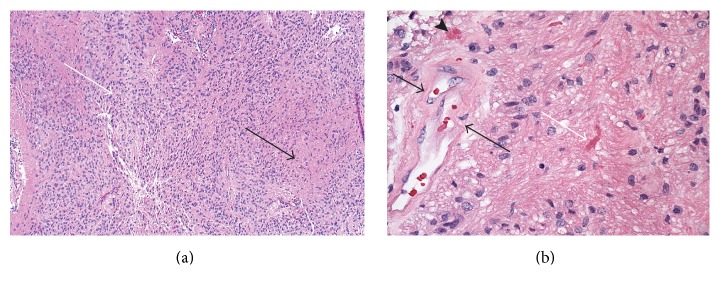
(a) H&E, mag ×100, cellular glial neoplasm composed of intermingled ependymal component (white arrow) and eosinophilic fibrillary component (black arrow). (b) H&E, mag ×400, both components of the lesion, pseudorosette on the left (black arrows) and fibrillary component with numerous Rosenthal fibers on the right, with the largest Rosenthal fiber in the center (black arrow). An eosinophilic body is seen on the left, above the vessel (arrowhead).

**Figure 4:**
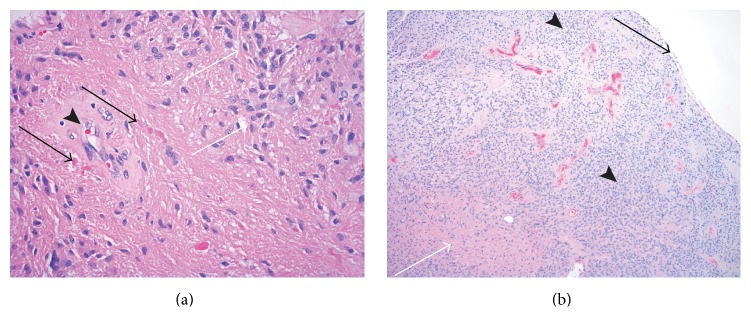
(a) H&E, mag ×400, bright eosinophilic deposits (black arrows) representing Rosenthal fibers in astrocytic component admixed with ependymal component (white arrows), adjacent to pseudorosette formation (arrowhead). (b) H&E mag ×40, pseudocapsule (black arrow) with exclusively ependymal component at periphery of the lesion (arrowheads); astrocytic component is in the center (white arrow).

Gross examination revealed an oval shape soft nodule with a smooth surface, which appeared encapsulated with a delicate membrane. Permanent sections showed cellular biphasic glial neoplasm: moderately cellular tumor with numerous perivascular pseudorosettes and less cellular areas with prominent eosinophilic fascicular component (Figure 3(a)). The cellular areas with pseudorosettes were consistent with ependymoma. The cells in the fascicular component were similar to the cells forming perivascular arrangements, but the cytoplasm was more eosinophilic and fibrillary. Many Rosenthal fibers were noted within the fascicular component, along with giant cell-like tumor cells and cystic change (Figure 3(b)). Focally, within the same pseudorosette, some cells show ependymal features, while others are more eosinophilic and fibrillary with Rosenthal fibers (Figure 4(a)). The fascicular component with Rosenthal fibers was representative of astrocytic lesion, reactive and/or tumor. Macro- and microcyst formation and Rosenthal fibers, along with giant tumor cells, which were present in fascicular component, are the known features of astrocytic tumor. There was no necrosis and the mitotic count was low. Interestingly, the periphery of the lesion consisted of ependymal component and central portion of the lesion had fibrillary and ependymal components intermingled (Figure 4(b)). These findings are consistent with a low grade glial lesion.

**Figure 5:**
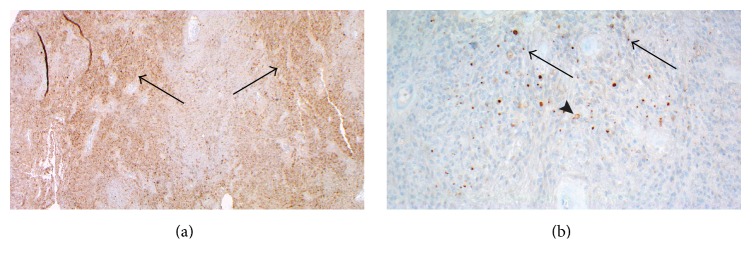
(a) Immunohistochemistry for synaptophysin, mag ×40, shows more intensive cytoplasmic staining in ependymal regions (arrows). (b) Immunohistochemistry for EMA, mag ×200, shows cytoplasmic, dot-like (arrows), and ring-like (arrowhead) cytoplasmic positivity in ependymal component.

The lesional cells, both components, showed strong, diffuse immunopositivity with glial fibrillary acidic protein (GFAP) and S-100 (more in fascicular component). Synaptophysin was staining stronger in the ependymal component (Figure 5(a)). The Ki-67 proliferation rate was low, around 1-2%, in both components. The epithelial membrane antigen (EMA) showed cytoplasmic dot-like positivity in many cells of ependymal component (Figure 5(b)).

In summary, the microscopic examination revealed low grade glial neoplasm, with histologic features of ependymoma and pilocytic astrocytoma, closely intermixed, and represented collision tumor.

The tumor was analyzed for mutation using a massively parallel (“next generation” or NGS) sequencing approach, performed by Foundation Medicine, INC, Cambridge, MA. This test assesses genomic alterations including base pair substitutions, insertions and deletions, and copy number alterations and select gene rearrangements for 315 cancer-related genes and introns from 28 genes often rearranged or altered in cancer, including NF1, NF2, IDH1, and TP53 [4]. The mutation in MUTYH G382D was identified in our sample. Normal tissue was not available for control testing. In addition, gene fusion testing was performed for the BRAF-KIAA1549 gene via FISH analysis, and the result was negative.

The patient had no postoperative complications. At one-month follow-up, she had resolution of lower extremity pain and tingling. At one-year imaging and clinical follow-up, patient had no evidence of tumor recurrence.

## 3. Discussion

Ependymoma (WHO grade II) is the most common intramedullary intradural spinal tumor followed by pilocytic astrocytoma. Although uncommon, spinal ependymomas (WHO grade II) have been described in an extramedullary, intradural location. To our knowledge, 23 such cases have been described [2, 3, 5–13]. The majority of these were in the thoracic spine, with four isolated to the lumbar spine [3, 6, 8]. The typical patient on presentation is a middle-aged female with spinal compressive symptoms of pain and/or myelopathy. All the previously described tumors were diagnosed as ependymomas. While a few reported cases showed higher grade components [9, 14], there were no cases of a collision/composite tumor.

Other extramedullary tumors of glial origin have been reported. Singh et al. described an astrocytoma in the conus region [15]. Kumar et al. reported a cystic pilocytic astrocytoma of the cauda equina, though that patient also had intracranial neoplasms and therefore likely had an underlying cancer-causing mutation such as NF1 or NF2 [16]. Both of these cases were primary extramedullary tumors with no intramedullary component.

Our case showed an extramedullary low grade glial tumor with histologic characteristics of an ependymoma and astrocytic lesion. Additionally, a fascicular component with Rosenthal fibers and cystic change was seen, consistent with pilocytic astrocytoma, and was intermixed with ependymoma component. This leads us to speculate the possibility that the lesion has dual origin: ependymal and astrocytic. We considered the option that the astrocytic component might represent piloid gliosis within a WHO grade II ependymoma. However, this was discounted as the astrocytic component was not focal or at periphery of the lesion, which one would expect for reactive change/piloid gliosis, but rather interspersed throughout and comprised at least half of the entire lesion. In addition, the presence of ependymal and fibrillary differentiation within the same pseudorosette further supports the hypothesis of compound tumor.

There are no absolute histologic criteria or immunohistochemical markers to differentiate piloid gliosis from pilocytic astrocytoma. Recent studies have shown that some pilocytic astrocytomas carry duplication at chromosome band 7q34 containing a BRAF-KIAA1549-gene fusion [17]. Although our specimen was negative for the KIAA1549-gene fusion transcript, the frequency of this is significantly lower in the adult population (<10% in above 40-patient populations). Therefore, the sensitivity of this diagnostic marker is weakened, and a negative FISH result does not rule out the possibility of the fascicular component of the lesion being pilocytic astrocytoma [18]. However, MUTYH G382D was identified by next-generation sequencing (NGS). To date, no association has been found between the MUTYH gene and glial neoplasia, though the G382D variant has been implicated in adenomatous polyposis [19]. Zadnik et al. identified several genes associated with astrocytoma, including CDK2NA, H2F3A, IDH1, NF1, TP53, ATRX, and PTEN [20]. All of these were tested by NGS and no mutations were found. Zadnik et al. also identified multiple genes associated with ependymoma, HOXB5, and PLA2G5, though neither of those were tested by NGS.

Several cases of mixed astrocytoma and ependymoma have been described, though none have been identified in the spine. Molnár and Hegedüs encountered an intraventricular ependymoma in the setting of a partially removed infiltrating astrocytoma in the frontal lobe [21]. However, this was felt to be a collision tumor between two distinct tumor origins. Kondziolka and Bilbao described a mixed ependymoma-astrocytoma in the parietal lobe, with features of a subependymoma but far from any ependymal lining [22].

There are case reports of ependymomas to have mesenchymal differentiation, for example, cartilage formation, which can be seen in adults and children [23, 24]. It is possible that we have a case where ependymal cells differentiate into astrocytic lineage.

## 4. Conclusion

To our knowledge, no cases of mixed ependymoma-astrocytoma have been reported. A tumor of glial origin should be included in the differential of an extramedullary, intradural mass, and based on our findings, a composite tumor should be considered in such a case.
